# Comprehensive characterization of human alveolar epithelial cells cultured for 28 days at the air-liquid interface

**DOI:** 10.1038/s41598-025-07219-8

**Published:** 2025-07-02

**Authors:** Ikuya Tanabe, Shinkichi Ishikawa

**Affiliations:** Scientific Product Assessment Center, R&D Group, Japan Tobacco Inc., 6-2 Umegaoka, Aoba-ku, Yokohama, Kanagawa 227-8512 Japan

**Keywords:** Alveolar epithelial cells, Air-liquid interface culture, Y-27632, A-83-01, CHIR99021, Lung surfactant, Tissue engineering, Stem-cell biotechnology

## Abstract

Lung epithelia are exposed to various substances in the atmosphere through breathing. To investigate the mechanisms and treatments for respiratory diseases caused by these substances, robust in vitro lung epithelial models are essential. This study aimed to develop an in vitro alveolar epithelial model using primary human pulmonary alveolar epithelial cells (HPAEpiCs). HPAEpiCs were cultured at an air-liquid interface (ALI) for 28 days in a medium supplemented with three small molecules: Y-27632, A-83-01, and CHIR99021. The characteristics of the ALI-cultured cells were then analyzed. Immunostaining revealed that many cells expressed alveolar type 2 (AT2) cell markers, such as surfactant protein B and prosurfactant protein C. Single-cell gene expression analysis further confirmed that the majority of the cells expressed genes reported to be highly expressed in AT2 cells. The apical surface of the ALI-cultured HPAEpiCs was covered with a liquid containing a variety of lipids and proteins known to be present in lung surfactant in vivo. Our data demonstrate that 28 days of ALI culture promoted the differentiation of HPAEpiCs into AT2 cells capable of secreting lung surfactant components. This ALI culture model containing differentiated AT2 cells could be valuable for investigating the mechanisms and treatments for respiratory diseases.

**Supplementary Information:**

The online version contains supplementary material available at 10.1038/s41598-025-07219-8.

## Introduction

The lungs play a critical role in gas exchange, acting as the primary site for oxygen absorption into the body and carbon dioxide excretion. Through the process of breathing, the lung epithelia are continuously exposed to various substances in the atmosphere, such as pollutants and pathogens. Consequently, the lung epithelia serve as an important line of defense against these harmful substances. When this line of defense becomes compromised, the harmful substances can cause lung diseases. As an example, pneumonia resulting from coronavirus infection during the COVID-19 pandemic has become a major global health issue in recent years^1^. This situation highlights the urgent need to investigate the causes, mechanisms, and treatments for respiratory diseases.

For research on respiratory diseases, robust in vitro human lung models that are reproducible, sustainable in long-term culture, and functionally relevant to the involved organ are essential^2^. Regarding in vitro human lung models, the use of primary human lung cells or lung cells derived from human induced pluripotent stem cells holds promise because lung cell lines derived from cancer patients generally lose some functions of normal healthy cells^3,4^. The initial goal during the development of in vitro human lung models is to create models that can reproduce specific functions of the lungs. Meanwhile, the creation of in vitro lung disease models through genetic manipulation could lead to better understanding of the disease mechanisms^5^. Furthermore, these disease models could be utilized to screen for therapeutic drugs.

The human lower respiratory tract can be divided into tracheal, bronchial, and alveolar regions^6^. In vitro models of the tracheobronchial epithelium have already been developed and are widely used. The tracheobronchial epithelium mainly consists of basal cells, ciliated cells, and goblet cells^6^. The function of the tracheobronchial epithelium is to remove inhaled foreign substances through mucociliary clearance to ensure clean air reaches the alveoli^7^. When human primary tracheobronchial epithelial cells are cultured at an air-liquid interface (ALI), they generate a mucociliary differentiated epithelium in vitro. Methods for ALI culture of human tracheobronchial epithelial cells have been detailed in several reports^8,9^.

The alveolar epithelium constitutes the majority of the lung surface area for gas exchange^10^. Despite its importance for lung function, in vitro models of the alveolar epithelium are lagging behind their tracheobronchial counterparts. This is attributable to the difficulty in culturing alveolar epithelial cells. The alveolar epithelium mainly consists of flat, thin alveolar type 1 (AT1) cells and cuboidal alveolar type 2 (AT2) cells^10^. AT1 cells are responsible for gas exchange, while AT2 cells secrete lung surfactant, promoting proper surface tension and protecting against inhaled pollutants and pathogens^10^. The low proliferative capacity of AT1 cells limits their use in vitro. AT2 cells have the capacity to proliferate and can differentiate into AT1 cells for tissue repair when the alveolar epithelium is damaged^11,12^. Thus, an effective approach for developing in vitro alveolar epithelial models involves proliferation of AT2 cells in vitro and induction of their differentiation into AT1 cells.

There are various approaches for the development of in vitro models of the alveolar epithelium comprising both AT1 and AT2 cells. Several in vitro human alveolar epithelial models using human primary alveolar epithelial cells or alveolar epithelial cells derived from human induced pluripotent stem cells have been reported. These models were developed by incorporating methods that maintain the proliferative capacity of AT2 cells in vitro, including use of feeder cells^13^spheroid culture^14^and optimized culture medium supplemented with small molecules or growth factors^15^. Several small molecules that can induce differentiation from AT2 cells to AT1 cells have also been incorporated^15–17^. The reported in vitro alveolar epithelial models include not only ALI culture models but also organoid models^18,19^.

Against this background, we have been developing an in vitro alveolar epithelial model through ALI culture of human pulmonary alveolar epithelial cells (HPAEpiCs) purchased from ScienCell Research Laboratories. Our previous research confirmed that HPAEpiCs can be passaged using medium supplemented with three small molecule inhibitors: Y-27632, A-83-01, and CHIR99021 (collectively abbreviated to YAC)^20^. Moreover, when HPAEpiCs were cultured at the ALI for 7 days in YAC-supplemented medium, they generated epithelium composed of cells expressing surfactant protein-B (SP-B) and prosurfactant protein-C (proSP-C), markers of AT2 cells^20^. In the present study, we investigated whether ALI-cultured HPAEpiCs can be cultured for a longer period while maintaining their differentiated state. For this, we conducted ALI culture of HPAEpiCs for 28 days, and subjected the cells to various analyses, including single-cell gene expression analysis, to comprehensively understand the differentiation status of the cells constituting the ALI-cultured HPAEpiCs. As an airway surface liquid (ASL) covering the surface of the HPAEpiCs was observed after 28 days of ALI culture, we conducted lipidomic and proteomic analyses on the ASL to determine the presence of lipids and proteins known to exist in lung surfactant in vivo.

## Results

### Epithelial barrier formation by HPAEpiCs

**Fig. 1:**
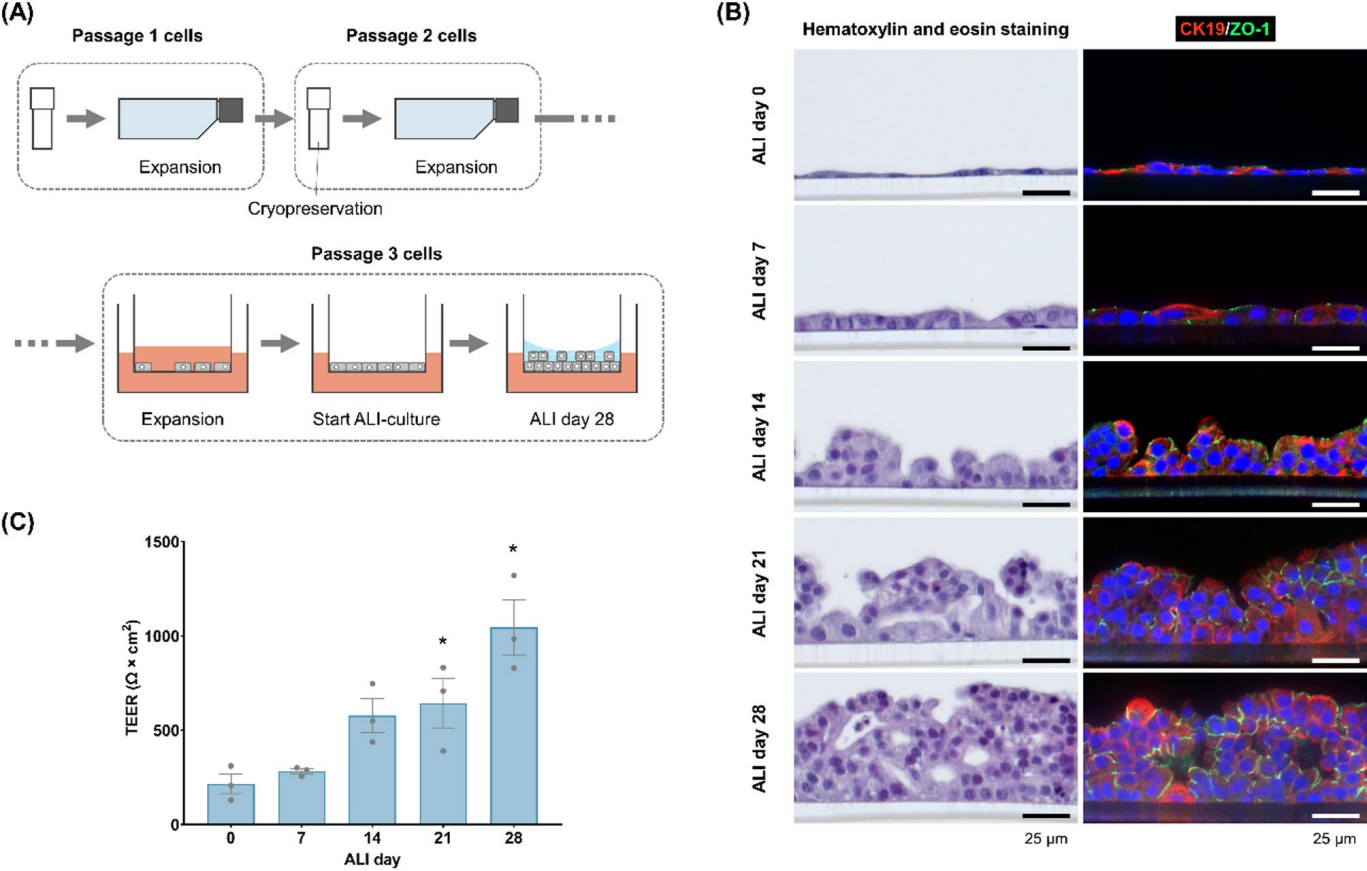
Analysis of the epithelial barrier function of HPAEpiCs at the ALI. (**A**) Schematic illustration of the cell culture procedure. HPAEpiCs at passage 1 were expanded and then stored at passage 2. HPAEpiCs at passage 3 were used for ALI culture. (**B**) Morphological analysis of HPAEpiCs on ALI days 0, 7, 14, 21, and 28. Some sections were stained with hematoxylin and eosin (left panels). Other sections were immunocytochemically stained with anti-cytokeratin 19 (CK19; epithelial marker) and zonula occludens-1 (ZO-1; tight junction marker) antibodies (right panels). (**C**) TEER measurements on ALI days 0, 7, 14, 21, and 28. The results are expressed as the mean ± standard deviation of three independent experiments (five transwell inserts per experiment). *p < 0.05, versus ALI day 0 by Dunnett’s multiple comparison test.

HPAEpiCs were expanded in YAC-supplemented medium, and passage 3 cells were cultured on transwell inserts at the ALI (Fig. 1A). The cultured HPAEpiCs were collected on ALI days 0, 7, 14, 21, and 28, and their morphology was examined. On ALI day 0, the cells appeared thin and flat, and were arranged in a monolayer (Fig. 1B). By ALI day 7, the cells had become cuboidal. The cells continued to proliferate, and formed a multilayer on the transwell insert by ALI day 28. The cells exhibited strong expression of cytokeratin 19, indicating that they were primarily composed of epithelial cells (Fig. 1B). Expression of the tight junction marker zonula occludens-1 increased with increasing duration of the ALI culture, with strong expression observed on ALI day 28 (Fig. 1B). Consistent with the zonula occludens-1 expression, the trans-epithelial electrical resistance (TEER) value increased with increasing duration of the ALI culture, reaching approximately 1000 Ω × cm^2^ on ALI day 28 (Fig. 1C). These findings indicate that HPAEpiCs formed an epithelial layer with a strong barrier function after 28 days of ALI culture.

### Analysis of the differentiation status of HPAEpiCs by qPCR

**Fig. 2:**
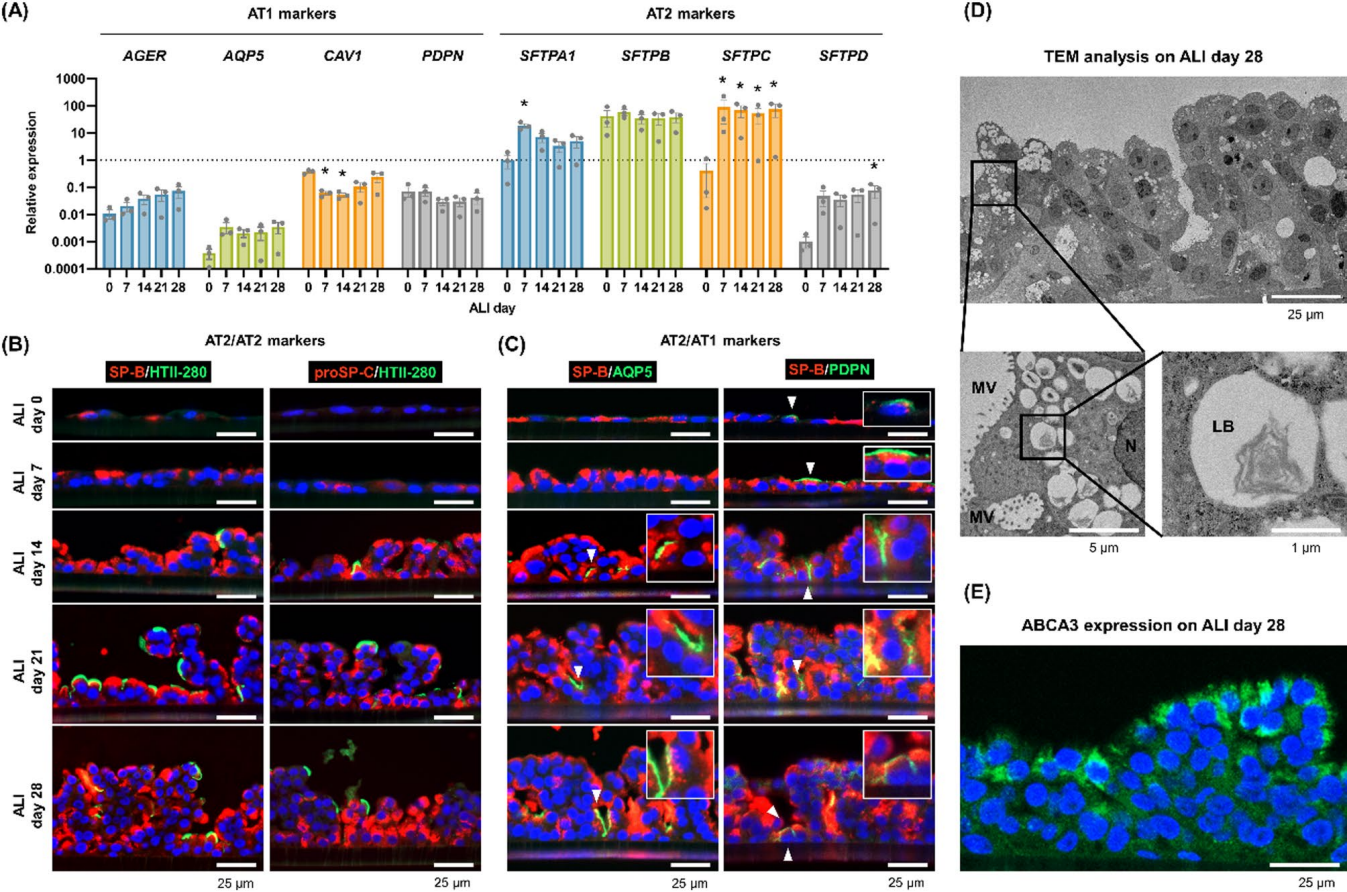
Analysis of the differentiation status of HPAEpiCs at the ALI. (**A**) Gene expression analysis of AT1 and AT2 markers by qPCR. The analysis was performed on mRNA samples obtained from HPAEpiCs on ALI days 0, 7, 14, 21, and 28. The gene expression of *SFTPA1* on ALI day 0 was used as a reference (relative expression set as 1). The results are expressed as the mean ± standard deviation of three independent experiments (two transwell inserts per experiment). *p < 0.05, versus ALI day 0 by Dunnett’s multiple comparison test. (**B**,**C**) Immunocytochemical analysis of HPAEpiCs on ALI days 0, 7, 14, 21, and 28. SP-B, proSP-C, and HTII-280 were analyzed as AT2 markers (**B**). AQP5 and PDPN were analyzed as AT1 markers (**C**). Arrowheads indicate cells positive for AQP5 or PDPN. (**D**) TEM analysis of HPAEpiCs on ALI day 28. LB, lamellar body; MV, microvilli; N, nucleus. (**E**) Immunostaining for lamellar bodies. The expression of ABCA3 was analyzed as a lamellar body marker.

To understand the differentiation status of the HPAEpiCs during ALI culture, qPCR analysis was conducted to examine the expression of several marker genes for AT1 cells (*AEGR*, *AQP5*, *CAV1*, *PDPN*)^21^ and AT2 cells (*SFTPA1*, *SFTPB*, *SFTPC*, *SFTPD*)^21^. The results were summarized using the expression level of *SFTPA1* on ALI day 0 as a reference (Fig. 2A). The expression of all AT1 marker genes was < 1 during the 28-day ALI culture, while the expression of the AT2 marker genes *SFTPA1*, *SFTPB*, and *SFTPC* was > 1 on ALI days 7, 14, 21, and 28 (Fig. 2A). The expression levels of the AT2 marker genes *SFTPA1*, *SFTPC*, and *SFTPD* increased significantly during the ALI culture (Fig. 2A). These findings indicate that the HPAEpiCs at the ALI strongly expressed AT2 marker genes.

### Analysis of the differentiation status of HPAEpiCs by immunocytochemistry

To confirm the validity of the qPCR data, immunocytochemical staining was performed for markers of AT1 cells and AT2 cells. First, we examined the expression of the AT2 markers SP-B, proSP-C, and HTII-280. SP-B was expressed in most cells on ALI day 0, and its expression was maintained until ALI day 28 (Fig. 2B). Expression of proSP-C was not observed on ALI day 0, but was subsequently detected on ALI day 7 and maintained until ALI day 28 (Fig. 2B). Expression of HTII-280 was first detected on ALI day 14 and maintained until ALI day 28 (Fig. 2B). Next, we examined the expression of the AT1 markers aquaporin 5 (AQP5) and podoplanin (PDPN) combined with the expression of SP-B (Fig. 2C). A small fraction of the cells expressed AQP5 or PDPN and also expressed SP-B (arrowheads, Fig. 2C).

The immunocytochemical findings indicated that the HPAEpiCs on ALI day 28 contained many cells expressing the AT2 markers SP-B, proSP-C, and HTII-280. Transmission electron microscopy (TEM) analysis was subsequently conducted to confirm the presence of microvilli and lamellar bodies, as typical organelles of AT2 cells^22^. The TEM images revealed that the cells covering the apical surface of the ALI-cultured HPAEpiCs had microvilli and numerous vacuoles containing lamellar bodies (Fig. 2D). Immunocytochemical staining revealed that ABCA3-positive lamellar bodies were localized on the apical surface cells of the ALI-cultured HPAEpiCs (Fig. 2E). These ABCA3-positive cells could be differentiated AT2 cells with lung surfactant-producing potential.

### Transcriptomic analysis of ALI-cultured HPAEpiCs

**Fig. 3:**
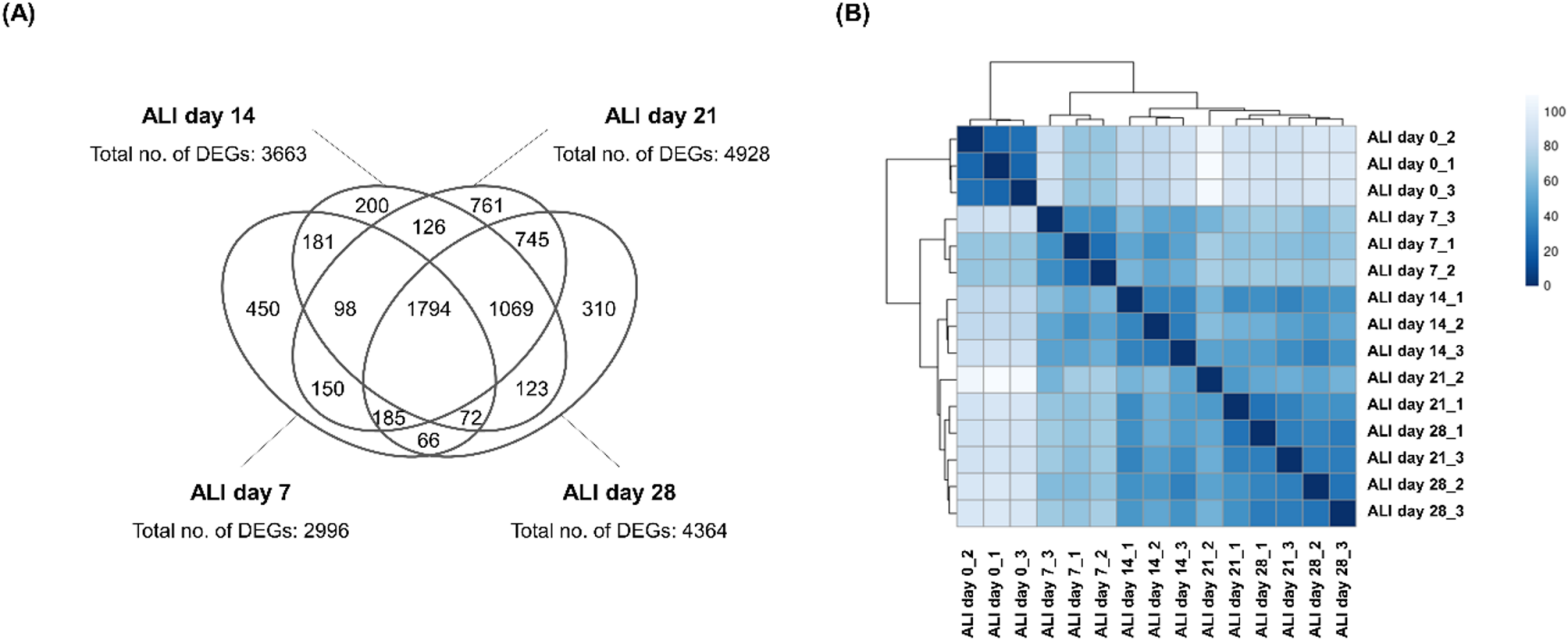
Analysis of the transcriptomic changes in HPAEpiCs during ALI culture using bulk RNA-seq. (**A**) Numbers of DEGs. DEGs were defined as genes with |log2 fold-change| >1.0 and adjusted *p*-value < 0.05. (**B**) Results of the cluster analysis. The analysis was performed on mRNAs derived from three transwell inserts prepared independently.

For further detailed analysis of the ALI-cultured HPAEpiCs, we applied bulk RNA sequencing (RNA-seq) to detect the transcriptomic changes in HPAEpiCs during the 28-day ALI culture. The numbers of differentially expressed genes (DEGs) were 2996 on ALI day 7, 3663 on ALI day 14, 4928 on ALI day 21, and 4364 on ALI day 28 (Fig. 3A). Since the maximum number of DEGs was observed on ALI day 21, the transcriptomic changes resulting from the initiation of ALI culture were considered to have stabilized by ALI day 28. These findings were supported by the results of a cluster analysis, which indicated that the data from ALI days 7 and 14 formed independent clusters, while the data from ALI days 21 and 28 were clustered together (Fig. 3B).

**Fig. 4:**
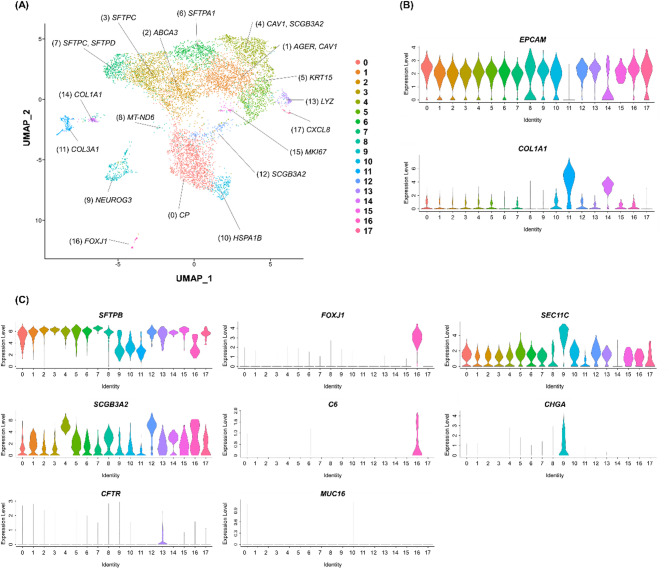
Deep characterization of HPAEpiCs on ALI day 28 using fixed RNA profiling. (**A**) UMAP plot of the fixed RNA profiling data for HPAEpiCs on ALI day 28. Eighteen clusters are summarized on the UMAP. The genes indicated in the figure are representative genes that showed significantly higher expression in each cluster compared with the other clusters (Bonferroni-corrected *p*-value < 0.05). (**B**) Violin plots showing the expression levels of representative marker genes. *EPCAM* was evaluated as a marker for epithelial cells, and *COL1A1* was evaluated as a marker for mesenchymal cells such as fibroblasts. (**C**) Violin plots showing the expression of genes associated with lower airway progenitor cells, ciliated cells, and neuroendocrine cells. *SFTPB*, *SCGB3A2*, and *CFTR* are known to be expressed by lower airway progenitor cells (cluster 13 cells). *FOXJ1* and *C6*, but not *MUC16*, are known to be expressed by lower airway ciliated cells (cluster 16 cells). *SEC11C* and *CHGR* are known to be expressed by some neuroendocrine cells (cluster 9 cells).

Since the transcriptomic changes in the ALI-cultured HPAEpiCs had stabilized by ALI day 28, we conducted a single-cell gene expression analysis for HPAEpiCs on ALI day 28 using fixed RNA profiling. A total of 18 clusters were detected (clusters 0–17, Fig. 4A). With the exception of cluster 11, all clusters strongly expressed the epithelial cell marker gene *EPCAM* (Fig. 4B). Cluster 11 may have contained contaminating mesenchymal cells, such as fibroblasts, because the cells in this cluster strongly expressed *COL1A1* (Fig. 4B). The top 10 most highly expressed genes in each cluster are summarized in Supplementary Table 1. All clusters, other than cluster 11, expressed typical genes reported to be highly expressed in AT2 cells, namely *CTSH*, *LPCAT1*, *NAPSA*, *PGC*, *RNASE1*, *SFTPA1*, *SFTPB*, *SFTPC*, *SLC34A2*, and *SLPI* (Supplementary Table 1)^23–25^.

Next, we extracted the genes that characterized each cluster. The top 10 genes showing significantly high expression in each cluster compared with the other clusters are summarized in Supplementary Table 2. The key genes for each cluster were selected from the list of top 10 genes for that cluster and are summarized in Table 1. Some key genes for each cluster are annotated in Fig. 4A, and their expressions are shown in Supplementary Fig. 1. Certain clusters expressed significantly high levels of alveolar epithelial marker genes. Specifically, clusters 2, 3, 6, and 7 expressed AT2 marker genes and clusters 1 and 4 expressed AT1 marker genes^21^ (Table 1). These clusters accounted for approximately 60% of the total cell number (Table 1). Other clusters exhibited significant expression of marker genes for different types of lung cells, including basal cells (cluster 5)^21^endocrine cells (cluster 9)^23,26^club cells (cluster 12)^21^fibroblasts (cluster 14)^27^and ciliated cells (cluster 16)^21,23,28^ (Table 1). Additional clusters showed significant expression of genes related to various biological functions, such as tissue defense (cluster 0)^29,30^mitochondrial function (cluster 8)^31^heat shock response (cluster 10)^32,33^host defense (cluster 13)^34^cell proliferation (cluster 15)^35^and inflammatory response (cluster 17)^36^ (Table 1).

**Table 1:** Genes exhibiting significant expression in each cluster and their functions.

Cluster no.	Cell counts	Cell ratio	Key genes	Function of key genes	References
0	1443	0.1674	*CP*, *FGA*, *FGB*, *FGG*, *SERPINA*	Tissue defense	^29,30^
1	1341	0.1556	*AGER*, *CAV1*	AT1 cell differentiation	^21^
2	1055	0.1224	*ABCA3*, *LAMP3*	AT2 cell differentiation	^21^
3	910	0.1056	*SFTPC*	AT2 cell differentiation	^21^
4	886	0.1028	*CAV1*, *SCGB3A2*	AT1/Club cell differentiation	^21^
5	591	0.0686	*KRT15*	Basal cell differentiation	^21^
6	575	0.0667	*SFTPA1*	AT2 cell differentiation	^21^
7	379	0.0440	*SFTPC*, *SFTPD*	AT2 cell differentiation	^21^
8	321	0.0372	*MT-CO2*, *MT-CO3*, *MT-ND6*	Mitochondrial function	^31^
9	249	0.0289	*CRYBA2*, *NEUROG3*, *PCSK1N*, *SEC11C*	Endocrine cell differentiation	^23,26^
10	224	0.0260	*ATF3*, *DNAJB1*, *NDRG1*, *HSPA1A*, *HSPA1B*,	Heat shock response	^32,33^
11	172	0.0200	*COL1A1*, *COL1A2*, *COL3A1*, *COL6A1*, *COL6A2*, *COL6A3*, *FN1*	Extracellular matrix production	^27^
12	142	0.0165	*SCGB3A2*	Club cell differentiation	^21^
13	116	0.0135	*LYZ*	Host defense	^34^
14	82	0.0095	*COL1A1*, *COL1A2*, *COL3A1*, *COL4A1*, *COL4A2*, *COL5A1*, *FN1*	Extracellular matrix production	^27^
15	48	0.0056	*HIST1H1A*, *HIST1H1B*, *HIST1H1C*, *HIST1H1D*, *MIK67*, *TK1*, *TOP2A*	Cell proliferation	^35^
16	45	0.0052	*C20orf85*, *CAPS*, *FOXJ1*, *TPPP3*	Ciliated cell differentiation	^21,23,28^
17	40	0.0046	*CCL20*, *CXCL1*, *CXCL2*, *CSCL3*, *CXCL8*, *NFKBIA*	Inflammatory response	^36^

Among the 18 detected clusters, the cells in cluster 13 were found to express *SFTPB*, *SCGB3A2*, and *CFTR* (Fig. 4B). The expression of these genes was reported as a typical characteristic of lower airway progenitor (LAP) cells^37^. LAP cells are known to differentiate into ciliated cells that express *C6*, but not *MUC16*^37^. LAP cells are also known to differentiate into neuroendocrine cells that express *CHGA*^37^. Cluster 16 cells expressed *C6* and the ciliated cell marker *FOXJ1*, while cluster 9 cells expressed *CHGA* and *SEC11C*, which was reported to be highly expressed in neuroendocrine cells^23,38^ (Fig. 4C). Therefore, the cluster 9 and 16 cells could be derived from cluster 13 cells. These findings indicate that the HPAEpiCs on ALI day 28 could contain LAP cells and their differentiated progeny.

### Lipidomic and proteomic analysis of ASL

**Fig. 5:**
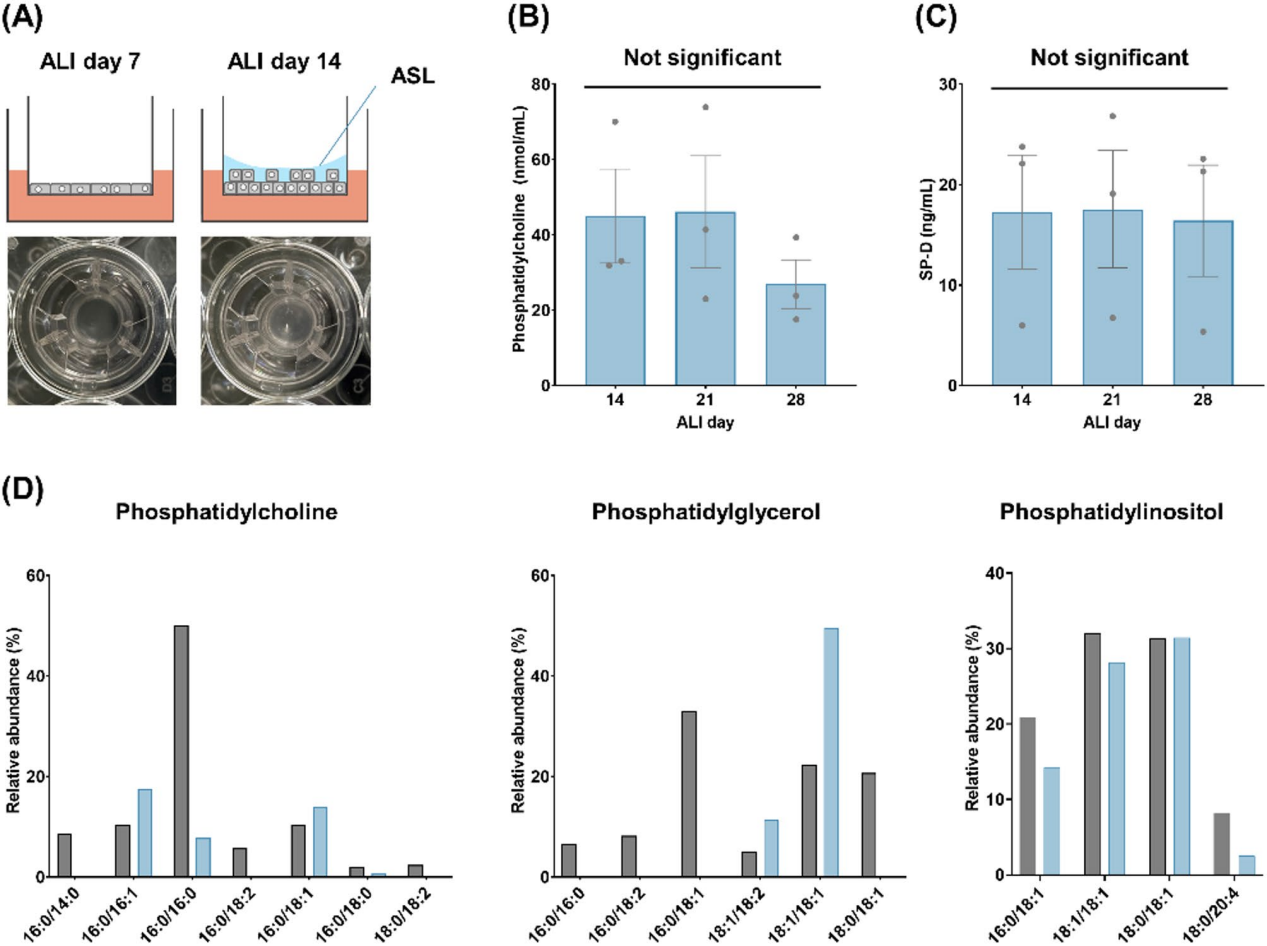
Results of the ASL analysis. (**A**) Pictures of a transwell with ASL covering the apical surface of the ALI-cultured HPAEpiCs. ASL was not observed on ALI day 7, but was observed on ALI day 14. (**B**,**C**) Concentrations of phosphatidylcholine (**B**) and SP-D (**C**) in the ASL. The results are expressed as the mean and standard deviation of three independent experiments (five transwell inserts per experiment). Not significant, *p* > 0.05, versus ALI day 14 by Dunnett’s multiple comparison test. (**D**) Comparison of the lipidomic data with in vivo values in the literature^39^. ASL collected on ALI day 28 was analyzed. The results for phosphatidylcholine, phosphatidylglycerol, and phosphatidylinositol are summarized. One pooled sample, derived from 30 transwell inserts prepared in two independent experiments, was used for the lipidomic analysis.

During the 28-day ALI culture, we observed a liquid covering the apical surface of the ALI-cultured HPAEpiCs. The ASL was first observed on ALI day 14 (Fig. 5A). We hypothesized that the ASL would contain lipids and proteins secreted by the differentiated AT2 cells with lamellar bodies (Fig. 2D, E). We collected the ASL on ALI days 14, 21, and 28, and analyzed the concentrations of phosphatidylcholine and surfactant protein-D (SP-D). Both factors were detected in the ASL from ALI day 14 to ALI day 28 (Fig. 5B, C).

For further detailed analysis of the ASL, we conducted a non-targeted lipidomic analysis. Among the lipids detected, phosphatidylcholine was the most abundant at approximately 60.97 pmol/µL, and accounted for approximately 50% of the total detected lipids in terms of molar ratios (Table 2). The detected lipids in each lipid class are summarized in Supplementary Table 3. The molar ratios of some lipids belonging to phosphatidylcholine, phosphatidylglycerol, and phosphatidylinositol in the in vitro ASL were compared with those in the in vivo lung surfactant^39^ (Fig. 5D).

**Table 2:** Major lipids detected in the ASL and their concentrations.

Lipid class	Concentration (pmol/µL)	Molar ratio (%)
Ceramide	4.93	4.15
Cholesterol esters	0.19	0.16
Diglycerides	2.59	2.18
Monoglycerides	0.05	0.04
Phosphatidylcholine	60.97	51.34
Phosphatidylethanolamine	14.43	12.15
Phosphatidylglycerol	3.47	2.92
Phosphatidylinositol	4.10	3.45
Phosphatidylserine	9.70	8.17
Sphingomyelin	17.82	15.01
Triglycerides	0.51	0.43

We also performed a proteomic analysis on the ASL. All of the detected proteins are summarized in Supplementary Table 4, while the top 20 proteins with the highest exclusive spectrum counts are summarized in Table 3. The most abundant protein was SP-B, followed by complement factor C3. In addition to complement factor C3, several typical serum proteins, such as complement factor H, complement factor I, apolipoprotein E, and alpha-1-antitrypsin, were also detected. These proteins were reported to exist in human bronchoalveolar lavage fluid^40^. We also detected carboxypeptidase M, which was reported to be useful for isolation of AT2 cell progenitors^41^and keratin type II cytoskeletal 8, which was reported to be expressed by differentiating AT2 cells^42^. Several proteins whose genes showed high expression in the fixed RNA profiling results were detected in the ASL (Supplementary Tables 1 and 2), including fibronectin (*FN1*), polymeric immunoglobulin receptor (*PIGR*), alpha-1-antitrypsin (*SERPINA1*), pro-cathepsin H (*CTSH*), ceruloplasmin (*CP*), and NPC intracellular cholesterol transporter (*NPC2*).

**Table 3:** Major proteins detected in the ASL and their exclusive spectrum counts.

	Identified proteins	Accession number	Alternate ID	Molecular weight	Exclusive spectrum count
1	Pulmonary surfactant-associated protein B	P07988	SFTPB	42 kDa	192
2	Complement C3	P01024	C3	187 kDa	163
3	Fibronectin	P02751	FN1	272 kDa	132
4	Complement factor H	P08603	CFH	139 kDa	131
5	Basement membrane-specific heparan sulfate proteoglycan core protein	P98160	HSPG2	469 kDa	88
6	Complement factor I	P05156	CFI	66 kDa	77
7	EGF-containing fibulin-like extracellular matrix protein 1	Q12805	EFEMP1	55 kDa	77
8	Cluster of Actin, cytoplasmic 1	P60709	ACTB	42 kDa	76
9	Polymeric immunoglobulin receptor	P01833	PIGR	83 kDa	76
10	Apolipoprotein E	P02649	APOE	36 kDa	70
11	Alpha-1-antitrypsin	P01009	SERPINA1	47 kDa	67
12	Carboxypeptidase M	P14384	CPM	51 kDa	67
13	Pro-cathepsin H	P09668	CTSH	37 kDa	67
14	Keratin, type II cytoskeletal 8	P05787	KRT8	54 kDa	55
15	Sulfhydryl oxidase 1	O00391	QSOX1	83 kDa	58
16	Ceruloplasmin	P00450	CP	122 kDa	57
17	Acid ceramidase	Q13510	ASAH1	45 kDa	55
18	NPC intracellular cholesterol transporter 2	P61916	NPC2	17 kDa	47
19	Lysosomal alpha-glucosidase	P10253	GAA	105 kDa	47
20	Dipeptidyl peptidase 4	P27487	DPP4	88 kDa	47

The protein identifications were filtered for a minimum of two peptides at 95% confidence and 99% protein confidence.

## Discussion

This study aimed to determine the characteristics of HPAEpiCs following long-term ALI culture in YAC-supplemented medium. Our previous study demonstrated that YAC-supplemented medium is useful for expansion of HPAEpiCs, showing expression of the AT2 cell markers SP-B and proSP-C after 7 days of ALI culture^20^. In the present study, HPAEpiCs were subjected to a longer duration of ALI culture. After 28 days of ALI culture, the HPAEpiCs had formed an epithelial layer with strong tight junctions. Various analyses indicated that the ALI-cultured HPAEpiCs contained a large number of cells expressing AT2 marker genes and proteins. The apical surface cells covering the ALI-cultured HPAEpiCs had lamellar bodies and microvilli on ALI day 28. These cells could be differentiated AT2 cells, and they secreted ASL following 28 days of ALI culture. The ASL contained several lipids and proteins known to exist in human lung surfactant. Taken together, these results suggest that 28 days of ALI culture in YAC-supplemented medium is appropriate for the differentiation of HPAEpiCs to AT2 cells.

We cultured HPAEpiCs at the ALI and analyzed the epithelial barrier function in the initial experiment. The HPAEpiCs formed a thin monolayered epithelium on ALI day 7, with a TEER value of approximately 250 Ω × cm^2^. These findings are consistent with our previous study using three lots of HPAEpiCs^20^. As the ALI culture continued, the HPAEpiCs proliferated further and formed a thick multilayered epithelium. The TEER value reached approximately 1000 Ω × cm^2^ on ALI day 28. This value is consistent with previous data for primary human AT2 cells and higher than those for other types of lung cells such as bronchial epithelial cells^6,43^. Therefore, the HPAEpiCs on ALI day 28 may have a barrier function similar to the actual in vivo alveolar epithelium.

To confirm the differentiation status of the cells composing the ALI-cultured HPAEpiCs, we conducted gene and protein expression analyses. The qPCR data indicated that the initiation of ALI culture promoted the expression of AT2 marker genes (*SFTPA1*, *SFTPC*, *SFTPD*) in HPAEpiCs. The immunocytochemical analysis indicated that most of the cells expressed SP-B from ALI day 0. The expression of two other AT2 markers, proSP-C and HTII-280, was induced by the initiation of ALI culture. HTII-280 is one of the markers for AT2 cells^44^and HTII-280-positive cells were reported to have a high capacity for generation of lung organoids^19^. Expression of HTII-280 was found to decrease during subculture of AT2 cells in previous studies^13,45^. Our data indicate that HTII-280-positive cells were absent on ALI day 0 and subsequently detected for the first time on ALI day 14. Thus, our culture conditions may reproduce some key environmental factors that induce the differentiation of AT2 cells with HTII-280 expression. We also confirmed that some SP-B-positive cells expressed the AT1 markers *AQP5* and *PDPN*, suggesting that these cells may be in an intermediate state of differentiation to AT1 cells. The presence of such cells was reported for an in vitro alveolar epithelial model derived from human pluripotent stem cells^46^. These findings indicate that long-term ALI culture promotes the differentiation of HPAEpiCs into AT2 cells, and that some cells are in an intermediate state for the differentiation process to AT1 cells^47^.

To capture the detailed differentiation status of the cells composing the HPAEpiCs on ALI day 28, we performed a single-cell gene expression analysis using fixed RNA profiling. The results indicated that the HPAEpiCs on ALI day 28 mainly consisted of cells with strong expression of AT2-related genes (Supplementary Table 1). Thus, the HPAEpiCs on ALI day 28 were predominantly composed of cell types sharing a gene expression profile with AT2 cells, with the exception of mesenchymal cell cluster 11. We also identified genes that showed significantly high expression in each cluster compared with the other clusters (Supplementary Table 2), and found that several clusters had significantly high expression of key genes associated with various lung cell types (Table 1). Specifically, cluster 9 represented neuroendocrine cells, while cluster 16 represented ciliated cells, with their progenitors potentially being in cluster 13 composed of cells expressing LAP signature genes such as *SFTPB*, *SCGB3A2*, and *CFTR*^37^. Meanwhile, both cluster 0 and cluster 12 cells expressed *CP* (Supplementary Fig. 1), a detoxification protein known to be expressed in a rare subset of AT2 cells (AT2-s cells)^23^. AT2-s cells were reported to potentially function as alveolar stem cells^23^. These findings suggest that the HPAEpiCs contained lower airway stem/progenitor cells, which may contribute to the diversity of the cell types detected by the single-cell gene expression analysis.

The cells in clusters 2, 3, 6, and 7 expressed the typical AT2 marker genes *ABCA3*, *SFTPC*, *SFTPA1*, and *SFTPD* more strongly than those in the other clusters. Therefore, the cells in these clusters could be fully differentiated AT2 cells. Specifically, the cells in cluster 2 could correspond to the ABCA3-positive cells identified by immunostaining that covered the apical surface of the HPAEpiCs on ALI day 28. The cells in clusters 1 and 4 strongly expressed the AT1 marker genes *AGER* and *CAV1* compared with those in the other clusters. These cells may be differentiating toward AT1 cells, observed as AQP5/PDPN-positive cells by immunostaining. The proportions of the cells in these clusters (clusters 2, 3, 6, and 7 expressing AT2 marker genes; clusters 1 and 4 expressing AT1 marker genes) were approximately 60%. Therefore, the HPAEpiCs on ALI day 28 were mainly composed of mature and differentiating alveolar epithelial cells.

The single-cell gene expression data aligned with the immunocytochemical findings, suggesting that the majority of the HPAEpiCs on ALI day 28 were AT2 cells. In the human lung in vivo, the ratio of AT2 to AT1 cells was found to be approximately 5:95 in terms of surface area^10^. However, in our immunocytochemical analysis, the AT1 markers AQP5 and PDPN were only expressed in limited areas. These findings may be attributed to the components of YAC (Y-27632, A-83-01, and CHIR99021) added to the culture medium, which play critical roles in maintaining the stemness of various cell types^48^. Because AT2 cells are considered local stem/progenitor cells, YAC may have contributed to the maintenance of AT2 cells as well as other types of stem/progenitor cells present in the HPAEpiCs, such as LAP cells. In particular, the Wnt activator CHIR99021 may have played a significant role, because Wnt signaling was identified as a key factor in the AT2 niche in vivo^49^. Meanwhile, the Wnt inhibitor XAV939 is known to promote the differentiation of AT2 cells into AT1 cells^17^. Therefore, incorporation of XAV939 into the culture medium for our method may lead to the establishment of an in vitro alveolar epithelial model that can reproduce the physiological balance between AT1 and AT2 cells more accurately.

Various results suggested that the ALI-cultured HPAEpiCs contained differentiated AT2 cells. Interestingly, we found that the surface of the ALI-cultured HPAEpiCs was covered with ASL on ALI day 14. One of the advantages of ALI culture is the accessibility to this ASL^50^. A previous study indicated that the ASL collected from ALI-cultured human bronchial epithelial cells contained a variety of mucin proteins that exist in the mucus layer on the bronchial epithelium in vivo^51^. Therefore, we hypothesized that the ASL of HPAEpiCs would contain lung surfactant components. The lipidomic analysis confirmed that the ASL contained several lipids known to be present in lung surfactant. Among the detected lipids, the proportion of phosphatidylcholine was very high, resembling the level observed in actual human lung surfactant^39^. Phosphatidylcholine 16:0/16:0 (dipalmitoylphosphatidylcholine [DPPC]) is the most abundant phosphatidylcholine in human lung surfactant^39^and it was detected in the ASL. However, the proportion of DPPC in the ASL (Fig. 5D) was smaller than that in lung surfactant in vivo^39^. We confirmed that the ASL collected from the ALI-cultured HPAEpiCs contained several lipids known to exist in lung surfactant in vivo. However, as evidenced by the results for DPPC in particular, the lipid composition of the ASL was insufficient to replicate the proportions of lipids found in human lung surfactant.

We also conducted a proteomic analysis of the ASL and a variety of proteins were found to be present. In particular, SP-B was the most abundant protein detected, consistent with the results of the qPCR, immunostaining, and fixed RNA profiling analyses. Pro-cathepsin H, which is involved in SP-B synthesis^52^was also detected, as were several proteins previously identified in lamellar bodies, including acid ceramidase^53^ and lysosomal alpha-glucosidase^54^. Complement proteins (complement factor C3, complement factor H, and complement factor I) and alpha-1-antitrypsin, which are involved in tissue defense, are known to be present in human lung surfactant. These proteins are typically produced in the liver and then transported to the lungs. However, previous studies have suggested that the lungs contain cells that are capable of synthesizing these proteins^55,56^. The ASL obtained from the ALI-cultured HPAEpiCs could contain proteins derived from cellular debris because carboxypeptidase M and keratin type II cytoskeletal 8, known to be expressed on the membrane of AT2 cells, were detected. However, our data indicated that the ASL of the ALI-cultured HPAEpiCs contained various proteins found in lung surfactant in vivo.

The lipidomic and proteomic analyses indicated that the ASL contained several lipids and proteins that are known to be present in lung surfactant in vivo. However, further research is necessary to determine the degree of similarity between the ASL and lung surfactant in vivo. Investigations into the expression levels and localizations of enzymes involved in lipid synthesis will help to clarify the ability of HPAEpiCs to synthesize lung surfactant. The use of labeled lipids will further clarify whether the lipid synthesis and recycling process is functioning appropriately in the ALI-cultured HPAEpiCs^57,58^. It will also be essential to determine whether the ASL exhibits surface-active properties comparable to those of lung surfactant in vivo^59^. These approaches will help to elucidate whether the AT2 cells in the ALI-cultured HPAEpiCs have the functional capacity to synthesize an ASL comparable to lung surfactant in vivo.

HPAEpiCs cultured at the ALI in YAC-supplemented medium could serve as a valuable tool for pulmonary research. The most distinctive advantage of our model is its simplicity, given that it requires the addition of only three small molecules to the culture medium. In contrast, previously reported models have involved complex procedures, such as media containing numerous molecules^15^spheroid cultures with gels^14^and multiple differentiation phases^18^. Another advantage of our model is the accessibility of the ASL secreted by AT2 cells, because abnormalities in surfactant production are associated with various lung diseases^60^. Moreover, disruptions in the proliferation and differentiation processes of AT2 cells contribute to the development of multiple lung diseases^61^. The ALI model was shown to effectively reproduce the transcriptomic changes occurring in vivo^62^. Future research using HPAEpiCs cultured at the ALI may help to elucidate the mechanisms by which external stimuli, such as pollutants and pathogens, affect AT2 cells and surfactant production, potentially contributing to a deeper understanding of various lung diseases.

Overall, our results indicated that 28 days of ALI culture of HPAEpiCs in YAC-supplemented medium yields differentiated AT2 cells with epithelial barrier integrity and potential for producing lung surfactant components. However, several aspects of the effects of YAC remain to be investigated. The first aspect is whether our findings can be reproduced using cells from different donors, as the present study was performed using cells from a single donor. A previous study indicated that HPAEpiCs have the potential to express AT2 markers when cultured at the ALI in YAC-supplemented medium for 7 days^20^. Other experiments using HPAEpiCs have employed small molecules comparable to YAC and growth factors to promote differentiation^14,15^. These studies suggest that the present findings may be applicable to cells from other donors. The second aspect is whether the effects of YAC are reproducible in adult-derived cells. Since the HPAEpiCs used in this study were fetal-derived, and fetal alveolar epithelial cells are known to exhibit different characteristics compared with adult-derived alveolar epithelial cells, this distinction is important. Commercially available human primary alveolar epithelial cells from Cell Biologics are a potential source of adult-derived alveolar epithelial cells, but these cells are known to lose their alveolar epithelial characteristics and transition to a basal cell phenotype during expansion in culture^63^. Therefore, it is essential to explore culture methods that can preserve the characteristics of adult-derived alveolar epithelial cells, and the use of YAC-supplemented medium may be a promising approach to address this issue.

## Conclusions

Our data indicated that HPAEpiCs can be cultured for 28 days at the ALI. Interestingly, this long-term ALI culture promoted HPAEpiCs to express the functions of AT2 cells, such as secretion of lung surfactant components. Since AT2 cells were reported to play a pivotal role in the development of various respiratory diseases, this differentiated AT2 cell model could be a useful tool for investigating the mechanisms and treatments for respiratory diseases.

## Methods

### ALI culture of HPAEpiCs

The HPAEpiCs were purchased from ScienCell Research Laboratories (Carlsbad, CA, USA) and passage 1 cells derived from a female at 20 weeks of gestation (can# 3200 and lot# 36278). The cells were cultured at 37 °C under 5% CO_2_ in Alveolar Epithelial Cell Medium (AEpiCM; ScienCell Research Laboratories) supplemented with YAC: 10 µM Y-27632, 0.5 µM A-83-01, and 3 µM CHIR99021 (all from Fujifilm Wako Pure Chemical Corporation, Osaka, Japan). The cells were seeded on collagen type I-coated T75 flasks for expansion (passage 1). On reaching semi-confluence, the cells were dissociated with trypsin and suspended in Cellbanker Type 1 (Zenogen Pharma, Fukushima, Japan) for cryopreservation (passage 2). For experiments, the cryopreserved cells were seeded on collagen type I-coated T25 flasks and allowed to reach semi-confluence. The cells were then dissociated with trypsin, suspended in YAC-supplemented AEpiCM at 2.5 × 10⁵ cells/mL, and applied to collagen type IV-coated 24-well transwell inserts at 125 µL/insert, resulting in a seeding density of 9.5 × 10^4^ cells/cm^2^ (passage 3). The bottom wells contained 700 µL of YAC-supplemented AEpiCM. After 3 days of culture, the medium on the apical side of the transwell inserts was removed to initiate ALI culture, and the medium in the bottom wells was replaced with 700 µL of YAC-supplemented AEpiCM. Medium changes were performed every 2 to 3 days. ALI culture was performed for 28 days.

### TEER measurement

TEER measurement was performed on ALI days 0, 7, 14, 21, and 28. The transwell inserts containing HPAEpiCs were transferred to a 24-well plate. Dulbecco’s Modified Eagle’s Medium was added to the apical inserts (200 µL/insert) and basolateral wells (700 µL/well). The TEER values (Ω × cm^2^) were measured using a Millicell ERS-2 (Merck Millipore, Burlington, MA, USA) and calculated as follows: TEER = (resistance of HPAEpiC insert – resistance of blank insert) × surface area of insert.

### Morphological analysis

HPAEpiCs on ALI days 0, 7, 14, 21, and 28 were washed with PBS, fixed with 4% paraformaldehyde in phosphate-buffered saline (PBS) at 4 °C, embedded in paraffin, and sectioned with a microtome at 4-µm thickness. After deparaffinization, some sections were stained with hematoxylin and eosin, and other sections were used for fluorescence immunocytochemical analysis. The antibodies used for the immunocytochemical analysis are summarized in Supplementary Table 5. Images were obtained with an AxioImager (Zeiss, Oberkochen, Germany).

### TEM analysis

HPAEpiCs on ALI day 28 were washed with PBS and fixed with 2% paraformaldehyde and 2% glutaraldehyde in PBS at 4 °C. The fixed HPAEpiCs were maintained in PBS containing 2% glutaraldehyde at 4 °C and sent to Tokai Electron Microscopy (Nagoya, Japan). The cells were then post-fixed with 2% osmium tetroxide. After dehydration and substitution, the cells were embedded in Quetol-812 resin (Nisshin EM, Tokyo, Japan) and sectioned with a microtome at 70-nm thickness. The sections were stained with uranyl acetate and lead stain solution for observation using a JEM-1400Plus (JEOL, Tokyo, Japan) with a CCD camera (EM-14830RUBY2; JEOL).

### RNA extraction and qPCR analysis

HPAEpiCs on ALI days 0, 7, 14, 21, and 28 were washed with PBS, and their RNA was extracted using an RNeasy kit (Qiagen, Hilden, Germany). The quality of the extracted RNA was assessed with a TapeStation 4200 (Agilent Technologies, Santa Clara, CA, USA). All samples had sufficient RNA quality (RNA integrity number > 7.0) and were applied to cDNA synthesis using SuperScript IV Reverse Transcriptase (Thermo Fisher Scientific, Waltham, MA, USA). The obtained cDNA was mixed with TaqMan Fast Advanced Master Mix (Thermo Fisher Scientific) and TaqMan Gene Expression Assays (Thermo Fisher Scientific) and analyzed by qPCR using a QuantStudio5 (Thermo Fisher Scientific). The TaqMan Gene Expression Assays used in this study detected *AGER* (Hs00542584_g1), *AQP5* (Hs00387048_m1), *CAV1* (Hs00971716_m1), *PDPN* (Hs00366766_m1), *SFTPA1* (Hs00831305_s1), *SFTPB* (Hs00167036_m1), *SFTPC* (Hs00161628_m1), and *SFTPD* (Hs01108490_m1). The relative expression levels were calculated using the ΔΔCt method using *18 S rRNA* (4319413E; Thermo Fisher Scientific) as an internal control. Expression of *SFTPA1* on ALI day 0 was used as a calibrator (relative expression = 1).

### Bulk RNA-seq analysis

RNA collected on ALI days 0, 7, 14, 21, and 28 was also subjected to RNA-seq analysis. The analysis was performed by Genble Inc. (Fukuoka, Japan). A DNA library was prepared using a TruSeq Stranded mRNA LT Sample Prep Kit (Illumina Inc., San Diego, CA, USA). Paired-end fastq sequence reads from each sample were obtained with a NovaSeq 6000 (Illumina Inc.). The obtained FASTQ data were converted to read counts using the Subio platform (ver. 1.24.5853; Subio Inc., Japan). The pipeline used for the conversion included fastp (ver. 0.22.0), HISAT2 (ver. 2.2.1), and StringTie (ver. 2.2.1). The FASTQ reads were aligned using HISAT2 and annotated using the human genome reference, Homo_sapiens GRCh38.112, from Ensembl (10.1093/nar/gkad1049). The count data were then processed using R (ver. 4.4.0) with RStudio (ver. 2023.12.1). The count data were converted to integer values, and genes with an average count below 15 were eliminated. Data normalization was performed using the DESeq2 method. The normalized data for each group on ALI days 7, 14, 21, and 28 were compared with the normalized data on ALI day 0 using the Wald test method. DEGs were defined as genes with |log2 fold-change| >1.0 and adjusted *p*-value < 0.05.

### Single-cell gene expression in fixed RNA profiling

HPAEpiCs on ALI day 28 were subjected to single-cell gene expression analysis by fixed RNA profiling. Briefly, HPAEpiCs were washed with PBS, incubated in 0.25% trypsin-EDTA for 5 min at 37 °C under 5% CO_2_, and detached from the inserts by pipetting. The detached HPAEpiCs were incubated for an additional 5-minute period and then separated by pipetting. Trypsinization was stopped by adding Dulbecco’s Modified Eagle’s Medium supplemented with 10% fetal bovine serum. The cell suspension was centrifuged, washed, and passed through a 40 μm Flowmi cell strainer (Bel-Art Products, Wayne, NJ, USA). The obtained cells were fixed with 4% formaldehyde in PBS at 4 °C for 20 h, and then treated with the Quenching Buffer and Enhancer contained in a Chromium Next GEM Single Cell Fixed RNA Sample Preparation Kit (10x Genomics, Pleasanton, CA, USA). The cells were sent to Genble Inc. for analysis. The details of the analysis are summarized in the Supplementary Information.

### Targeted ASL analysis

ASL samples were collected on ALI days 14, 21, and 28. Approximately 5–10 µL of ASL was obtained from each transwell insert. The samples from five transwell inserts were pooled and centrifuged for 5 min at 10,000 rpm and 4 °C to eliminate cellular debris. The resulting ASL samples were analyzed using a Phosphatidylcholine Assay Kit (Sigma-Aldrich, St. Louis, MO, USA) and a Human SP-D Quantikine ELISA Kit (R&D Systems, Minneapolis, MN, USA).

### Non-targeted ASL analysis

ASL samples were collected from 15 transwell inserts on ALI day 28, and centrifuged for 5 min at 10,000 rpm and 4 °C to eliminate cellular debris. Lipidomic analysis was performed by Lipidome Lab Inc. (Akita, Japan) with a non-targeted lipidome scan package. Lipids were extracted using a modified Bligh & Dyer method. The specimens were dried under nitrogen gas, dissolved in methanol, and applied to LC-ESI-MS/MS analysis using a Dionex Ultimate 3000 UPLC system (Thermo Fisher Scientific) with an L-column3 C18 metal-free column (100 × 2.0 mm i.d.; 2.0 μm; CERI, Tokyo, Japan) coupled to a Q Exactive Plus (Thermo Fisher Scientific). For peak selection, estimates for the lipid species and alignments between the samples were analyzed using the lipid identification software Lipid Search (Mitsui Information, Tokyo, Japan). The proteomic analysis was performed by APRO Science (Tokushima, Japan) with an LC-MS/MS shotgun analysis. Briefly, the ASL samples were reduced with dithiothreitol, alkylated with iodoacetamide, and digested with trypsin. The samples were then applied to LC-MS/MS analysis using an EASY-nLC 1200 system (Thermo Fisher Scientific) with an EASY-spray column (15 cm × 75 μm i.d.; 3-µm particles; 100-Å pore size; Thermo Fisher Scientific) coupled to a Q Exactive Plus. Data were analyzed with Scaffold 5 (Proteome Software, Portland, OR, USA) against the SwissProt database at APRO Science.

### Statistical analysis

Three independent experiments were carried out for TEER measurement, qPCR analysis, and targeted ASL analysis. Each experiment was performed with five transwell inserts for TEER measurement, two transwell inserts for qPCR analysis, and five transwell inserts for targeted ASL analysis. The results were expressed as the mean and standard deviation of the three independent experiments. Statistical analyses were performed using GraphPad Prism software (version 9.3.1; Dotmatics, Boston, MA, USA). The results were considered significant for values of *p* < 0.05 versus the control condition by Dunnett’s multiple comparison test. RNA-seq analysis was performed on samples derived from HPAEpiCs prepared in three independent experiments, each with one transwell insert. Fixed RNA profiling was performed on one pooled sample derived from 20 transwell inserts of HPAEpiCs prepared in one experiment. Non-targeted ASL analysis was performed on one pooled sample derived from 30 transwell inserts of HPAEpiCs prepared in two independent experiments (15 transwell inserts per experiment).

## Electronic supplementary material

Below is the link to the electronic supplementary material.

Supplementary Material 1

Supplementary Material 2

Supplementary Material 3

## Data Availability

The bulk RNA-seq and fixed RNA profiling data are available in ArrayExpress under the accession numbers E-MTAB-14793 and E-MTAB-14782, respectively.
