# Supplementary material for: Comprehensive characterization of human alveolar epithelial cells cultured for 28 days at the air-liquid interface
Source: Sci Rep. 2025 Jul 2;15:22995. doi: 10.1038/s41598-025-07219-8 (PMC12219739; doi:10.1038/s41598-025-07219-8)
Supplement: Supplementary file 1 — Supplementary Material 1 [file 41598_2025_7219_MOESM1_ESM.docx]

**Single-cell gene expression by fixed RNA profiling**

**Methods**

**Library preparation**

Cells were fixed and stored using a Chromium Next GEM Single Cell Fixed RNA Sample Preparation Kit (10x Genomics, Pleasanton, CA, USA). Cell barcoding, amplification, and library construction were performed using a Chromium Fixed RNA Reagent Kit (10x Genomics), in accordance with the manufacturer’s protocol. A single-cell suspension was obtained by filtering the cell suspension through a 30-μm cell strainer after hybridization with a Chromium Human Transcriptome Probe Set (version 1.0.1). Isolated single cells were loaded onto a 10x Chromium X (10x Genomics), barcoded, and pre-amplified to construct the sequencing library.

**Sequencing**

The library was sequenced on a DNBSEQ-G400 platform (MGI Tech, Shenzhen, China) with the following sequencing parameters: 28 bp for read 1; 100 bp for read 2.

**Data processing**

The sequenced reads were subjected to demultiplexing, alignment, barcode counting, UMI counting, and filtering using Cell Ranger (version 7.1.0; 10x Genomics). The human genome (GRCh38-2020-A) was used as a reference to align the reads.

**Data analysis and visualization**

The following data analysis was performed in R (version 4.1.0). Ambient RNA counts were estimated and removed by SoupX. The SoupX-adjusted counts were applied to the differential gene expression analysis and visualization with the R package Seurat (version 4.0.3). Genes expressed in <3 cells were eliminated. Potential doublet cells were filtered out using DoubletFinder. Cells expressing <200 genes or >7,500 genes were removed, as were cells with >5% mitochondrial-associated genes among their expressed genes and those with >30,000 UMI counts. The UMI counts were log-normalized (scale factor = 10,000). We regressed out the S scores, G2/M scores, and percentages of mitochondrial-associated genes during the data scaling. Dimensionality reduction was performed using a principal component analysis. The first 60 principal components were used to cluster the cells based on the shared nearest neighbor algorithm, and the cells were visualized in two dimensions by Uniform Manifold Approximation and Projection. For differential gene expression analysis, the likelihood ratio test was performed using the MAST package, and the calculated *p*-values were adjusted for multiple comparisons using a Bonferroni correction.
