# Supplementary material for: Comprehensive characterization of human alveolar epithelial cells cultured for 28 days at the air-liquid interface
Source: Sci Rep. 2025 Jul 2;15:22995. doi: 10.1038/s41598-025-07219-8 (PMC12219739; doi:10.1038/s41598-025-07219-8)
Supplement: Supplementary file 3 — Supplementary Material 3 [file 41598_2025_7219_MOESM3_ESM.docx]

**
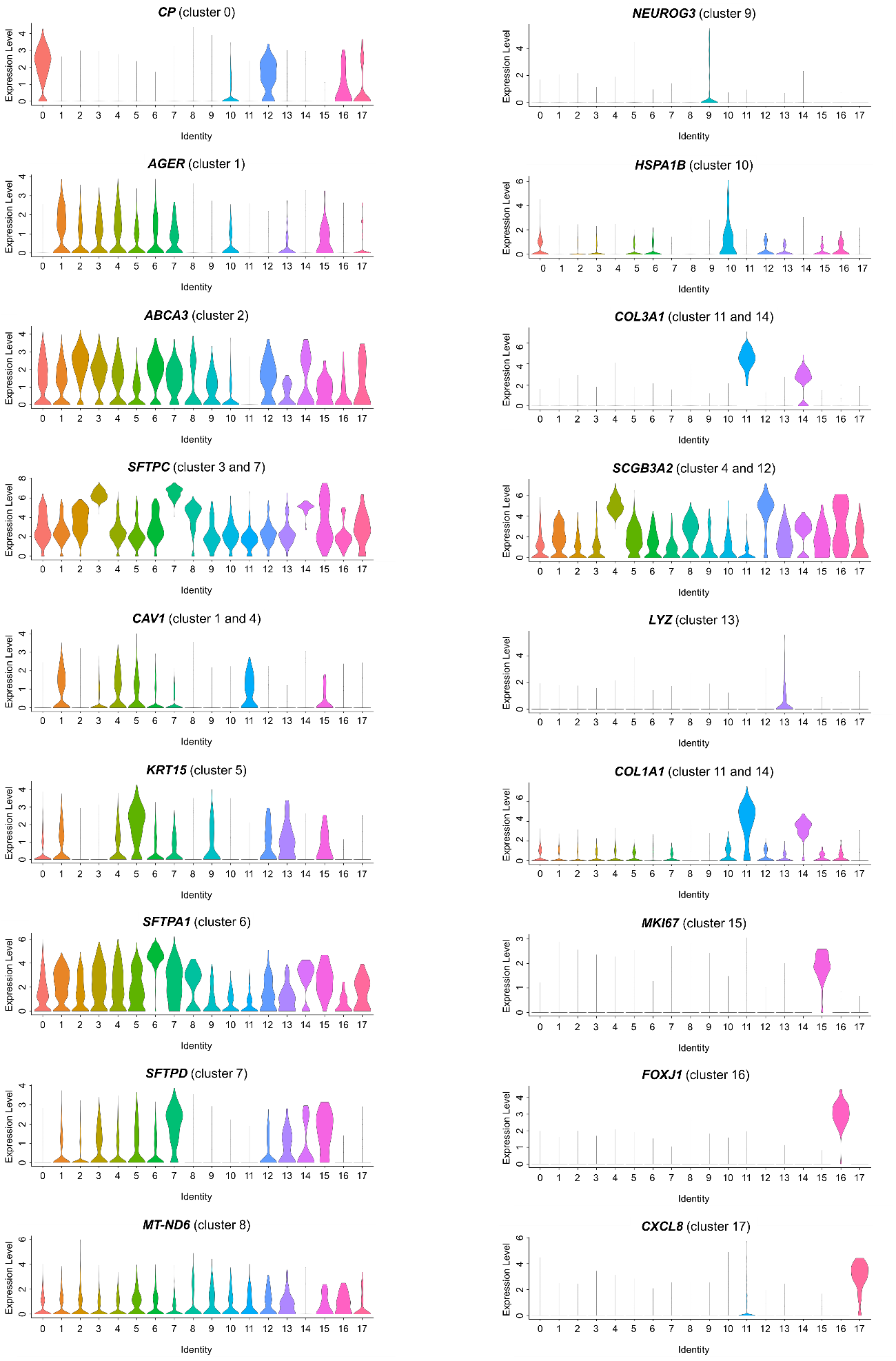
**

**Supplementary Figure 1. Violin plots showing the expression levels of representative genes in the 18 clusters.** The representative genes were selected from those that exhibited significantly higher expression in each cluster compared with the other clusters.
